# Prevalence of Eosinophilic Esophagitis in Patients With Gastroesophageal Reflux Symptoms

**DOI:** 10.7759/cureus.90649

**Published:** 2025-08-21

**Authors:** Sabin Rajbhandari, Dibas Khadka, Kumud Bhattarai, Mukesh S Paudel

**Affiliations:** 1 Department of Gastroenterology, National Academy of Medical Sciences, Bir Hospital, Kathmandu, NPL

**Keywords:** eosinophilic esophagitis (eoe), esophago-gastro-duodenoscopy, gastroesophageal reflux symptoms, prevalence, proton-pump inhibitors (ppi)

## Abstract

Background: Eosinophilic esophagitis (EoE) is a chronic, immune- or antigen-mediated inflammatory condition of the esophagus, which is characterized by eosinophil-predominant infiltration and resulting esophageal dysfunction. In adults, it typically presents with dysphagia and food impaction. In children, common symptoms include vomiting, feeding difficulties, and abdominal pain. Diagnosis is established in symptomatic individuals by esophagogastroduodenoscopy with esophageal biopsy, demonstrating at least 15 eosinophils per high-power field (HPF) in the esophageal mucosa. Despite increasing recognition of EoE globally, data from low- and middle-income countries (LMICs), including Nepal, remain scarce.

Objectives: This study was conducted to find the prevalence of EoE in patients with symptoms of gastroesophageal reflux (GER) in Nepal.

Materials and methods: A cross-sectional study was carried out at the tertiary care level central hospital in Nepal from January 2024 to August 2024, among a total of 60 participants with symptoms of GER. All participants underwent an esophagogastroduodenoscopy (EGD), and esophageal biopsies were taken to check for the presence of eosinophilic esophagitis (EoE). Data on demographic and clinical characteristics, endoscopic findings, peripheral blood absolute eosinophil count, and history of proton pump inhibitors (PPI) use were obtained. Statistical analysis was done using IBM SPSS Statistics for Windows, version 21 (IBM Corp., Armonk, NY, USA).

Results: EoE was identified in four patients (6.67%) based on histopathological examination of esophageal biopsy samples with no gender predilection and was found predominantly in participants aged 40 or more. The findings suggest the consideration of EoE as a diagnostic possibility in patients with GER symptoms in Nepal.

## Introduction

Eosinophilic esophagitis (EoE) refers to a chronic, immune- or antigen-mediated esophageal disease described by symptoms correlated to eosinophil-predominant inflammation and esophageal dysfunction. It was first termed by Landres et al. in 1978 and progressively recognized as a chronic inflammatory esophageal disease among all types of populations globally, in a very short period [1,2].

Food-based antigens mostly mediate this type of disease. Clinically, the following components define EoE: dysphagia, food impaction in adults, vomiting, feeding problems, and abdominal pain in children; esophageal mucosal eosinophilia of a minimum of 15 eosinophils per high-power field (HPF). The conditions, such as gastroesophageal reflux disease (GERD), must be ruled out while diagnosing EoE, as they show similar symptoms [3,4]. Eosinophils are found over the gastrointestinal (GI) tract distal to the squamous esophagus under physiological conditions [5]. Various conditions, such as eosinophilic esophagitis (EoE), gastroenteritis, GERD, eosinophilic gastritis, colitis/Crohn’s disease with esophageal involvement, esophageal motility disorders such as achalasia, fungal and viral infections, and hypereosinophilic syndrome, are linked with the esophageal eosinophilia (EE) [6,7].

Gastroesophageal reflux (GER) is not considered a standalone disease. In contrast, GERD is classified as a disease that occurs when the normal mechanism that prevents the reflux of stomach contents fails. GERD can cause symptoms such as acid regurgitation, chest pain, and heartburn. In most cases, an endoscopy does not reveal any visible damage. However, in some patients, conditions such as erosive esophagitis, Barrett's esophagus, or peptic strictures may be observed. Chest pain or signs of extra-esophageal manifestations related to the lungs, ears, nose, or throat may also be observed [6,7]. In the US, 8.9 million outpatient clinic visits happened in 2009 for GERD, indicating it is the primary diagnosis for GI syndromes [8].

Studies on EoE are primarily based on case series and reports, and data regarding EoE are scarce in low- and middle-income countries (LMICs) [9-13]. In this regard, EoE epidemiology needs to be well documented in such nations. It is crucial to determine whether the low reporting in LMICs accurately reflects the rarity of EoE or if it is underreported. There are no data available in Nepal on EoE. This study was conducted on patients with GER symptoms to diagnose EoE in this population.

## Materials and methods

Study design and setting

This was a hospital-based cross-sectional study conducted at the Department of Gastroenterology, Bir Hospital, National Academy of Medical Sciences (NAMS), Kathmandu, Nepal, from January 2024 to August 2024. Bir Hospital is a tertiary-level hospital located in the capital city of Nepal, where patients from across the country visit for specialty care.

Study participants

All adult patients (> 18 years) with GER symptoms such as acid regurgitation, chest pain, and heartburn who consented to Esophagogastroduodenoscopy (EGD) and biopsy were included in the study. Patients with a previous diagnosis of eosinophilic esophagitis, drug-induced esophagitis, esophageal malignancy, infectious esophagitis, Crohn’s disease, coagulopathy, esophageal varices, thrombocytopenia, and proton pump inhibitor (PPI) usage within the last four weeks were excluded from the study.

Sample size

The sample size was calculated using the prevalence formula, taking p 0.15, and a 10% margin of error at a 95% confidence interval [14].

$N = \frac{Z^{2}pq}{e^{2}}$

Where, Z = 1.96 at a 95% confidence interval, p = 0.15 (Prevalence), q = 1-p = 0.85, e = 0.1 (margin of error).

The sample size obtained was 49. Hence, taking a 10% dropout rate, the final sample size (N) was calculated to be 54, and we considered a total of 60 patients.

Data collection

Adult patients (>18 years) with GER symptoms presenting to the Gastroenterology OPD at Bir Hospital, from January 2024 to August 2024, were screened. All patients who met the inclusion criteria underwent EGD. The Los Angeles (LA) classification was used to describe and grade erosive esophagitis [15]. Barrett’s esophagus, esophageal ulcers, diverticula, or hiatus hernia were also noted. Endoscopists were not blinded to the patient’s clinical history. Using standard biopsy forceps, a minimum of six biopsies from pre-defined upper (5 cm below the upper esophageal sphincter) and lower end of the esophagus (5 cm above the gastroesophageal junction), and any other endoscopically visible abnormal mucosa were obtained. Any interventions or biopsies were performed at the discretion of the endoscopist. Data on demographic and clinical characteristics, endoscopic findings, peripheral blood absolute eosinophil count, and history of PPIs usage were obtained and analyzed. EoE was diagnosed if the esophageal eosinophil count was more than 15 per HPF. All relevant data for each study subject were collected and recorded in a pre-designed data collection sheet.

Statistical analysis

A structured proforma was used to collect data. Statistical analysis was done using IBM SPSS Statistics for Windows, version 21 (IBM Corp., Armonk, NY, USA). Quantitative and qualitative variables were compared using the mean ± standard deviation and proportion, respectively. Continuous data were analyzed using Student’s t-tests, while categorical data were analyzed with the chi-square test. The results were presented in tables and diagrams. The point estimate was calculated at a 95% confidence interval.

Ethical considerations

Ethical clearance was obtained from the Institutional Review Board (IRB), National Academy of Medical Sciences, Bir Hospital, Kathmandu (Reference No.: 1030/2080/81, dated December 28, 2023). All participants provided written informed consent before their inclusion in the study, with assurances of confidentiality.

## Results

Among 60 consecutive patients with symptoms of GER who were screened, 30 (50%) were above 40 years of age, and most of the patients (39, 65%) were female participants. All had absolute eosinophilic counts (AEC) within normal limits. Giardiasis was observed in four of the participants, but not in patients with EoE. Reflux esophagitis was seen in six (10%) (LA-A=3, LA-B=1, LA-C=1, LA-D=1) patients, whereas esophageal ulcer was seen in two (3.33%) patients on EGD, along with one case of Barrett’s esophagus during the EGD (Table 1).

**Table 1:** Sociodemographic data and clinical characteristics SN: serial number; EGD: esophagogastroduodenoscopy; LA: Los Angeles classification

SN	Particulars	Number (N)	Frequency (%)
1	Age		
	Below 40	30	50
	40 and above	30	50
2	Sex		
	Male	21	35
	Female	39	65
3	EGD		
	Normal	51	85
	Reflux esophagitis	6	10
	LA-A	3	5
	LA-B	1	1.67
	LA-C	1	1.67
	LA-D	1	1.67
	Barrett’s esophagus	1	1.67
	Esophageal ulcer	2	3.33
4	Stool routine examination		
	Normal	56	93.33
	Abnormal (Giardiasis)	4	6.67

**Figure 1:**
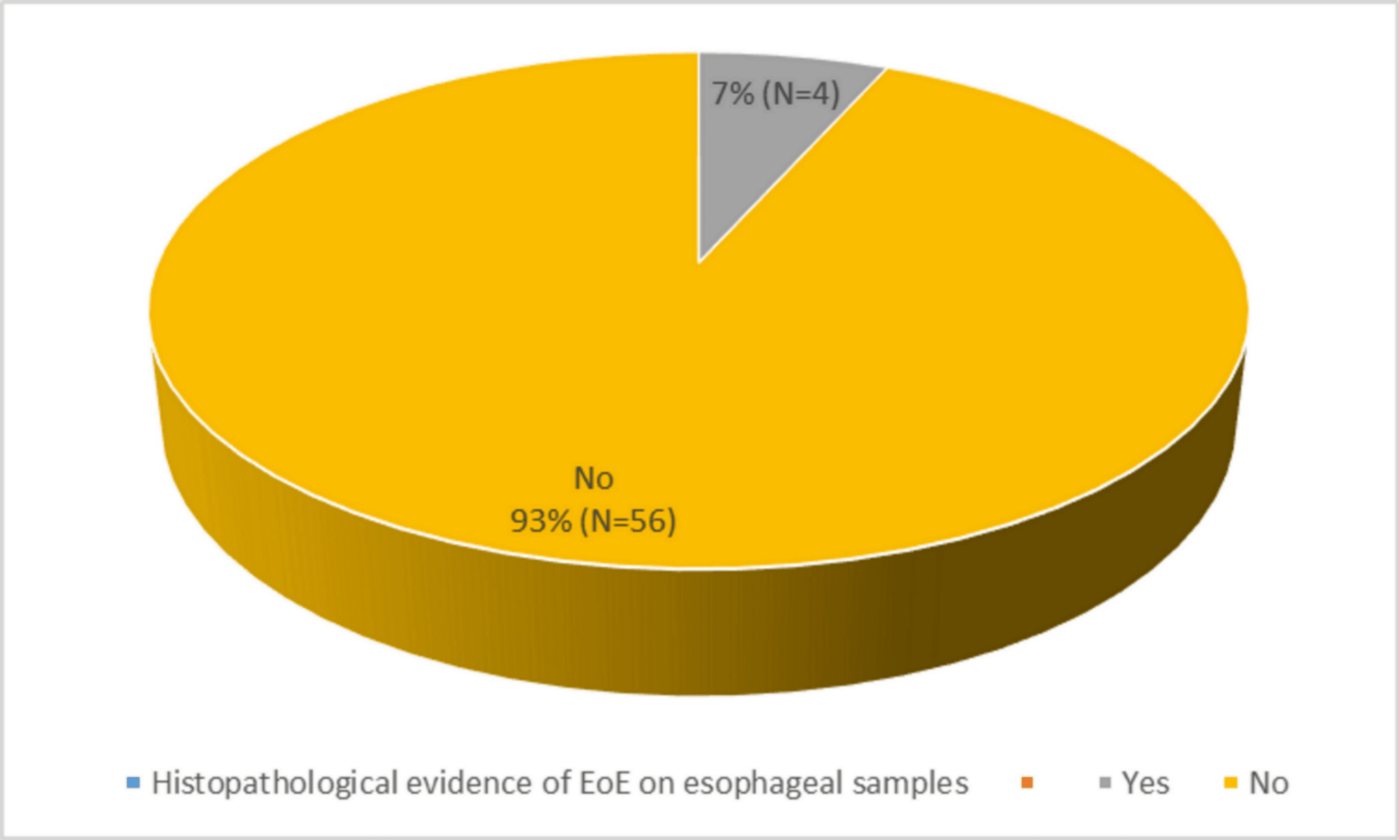
Biopsy findings of the patients EoE: eosinophilic esophagitis

**Figure 2:**
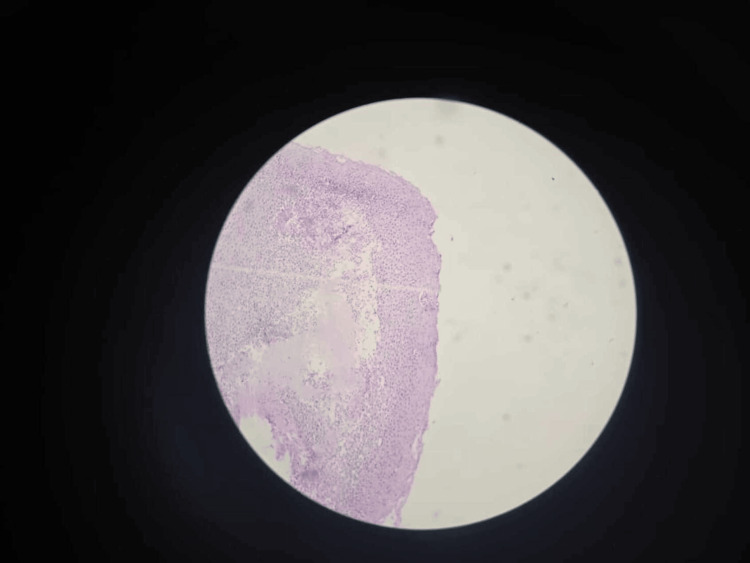
Esophageal mucosa with an infiltrate of >100 eosinophils per high power field, consistent with EoE (H&E stain under 10x magnification) EoE: eosinophilic esophagitis; H&E: hematoxylin and eosin

**Figure 3:**
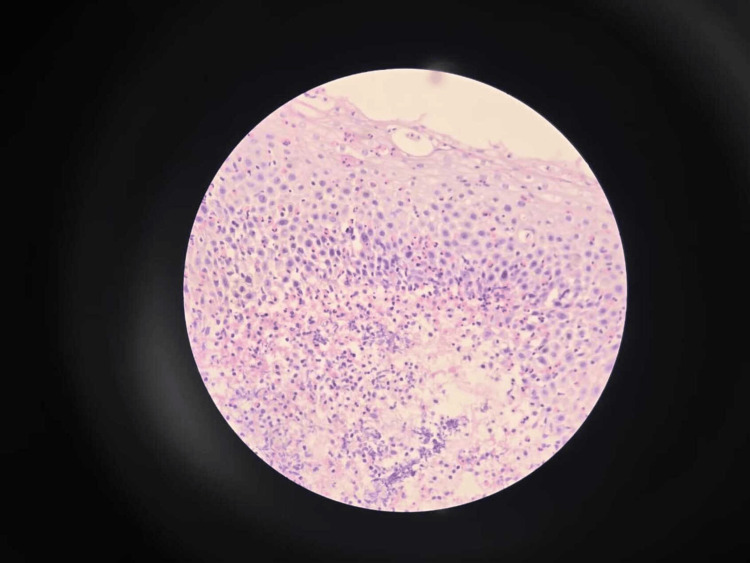
Esophageal mucosa with an infiltrate of >100 eosinophils per high power field, consistent with EoE (H&E stain under 40x magnification) EoE: eosinophilic esophagitis; H&E: hematoxylin and eosin

EoE was evident in four (6.67%) patients on histopathological examination of the esophagus, demonstrating eosinophilia on microscopy along with Barrett’s esophagus in one (1.67%) participant (Figures 1-3).

**Figure 4:**
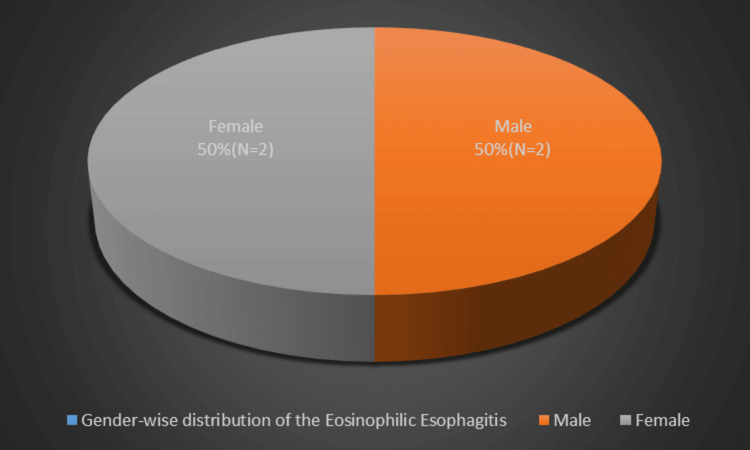
Gender-wise distribution of the eosinophilic esophagitis

Participants with diagnosed EoE showed equal sex distribution (Figure 4).

**Figure 5:**
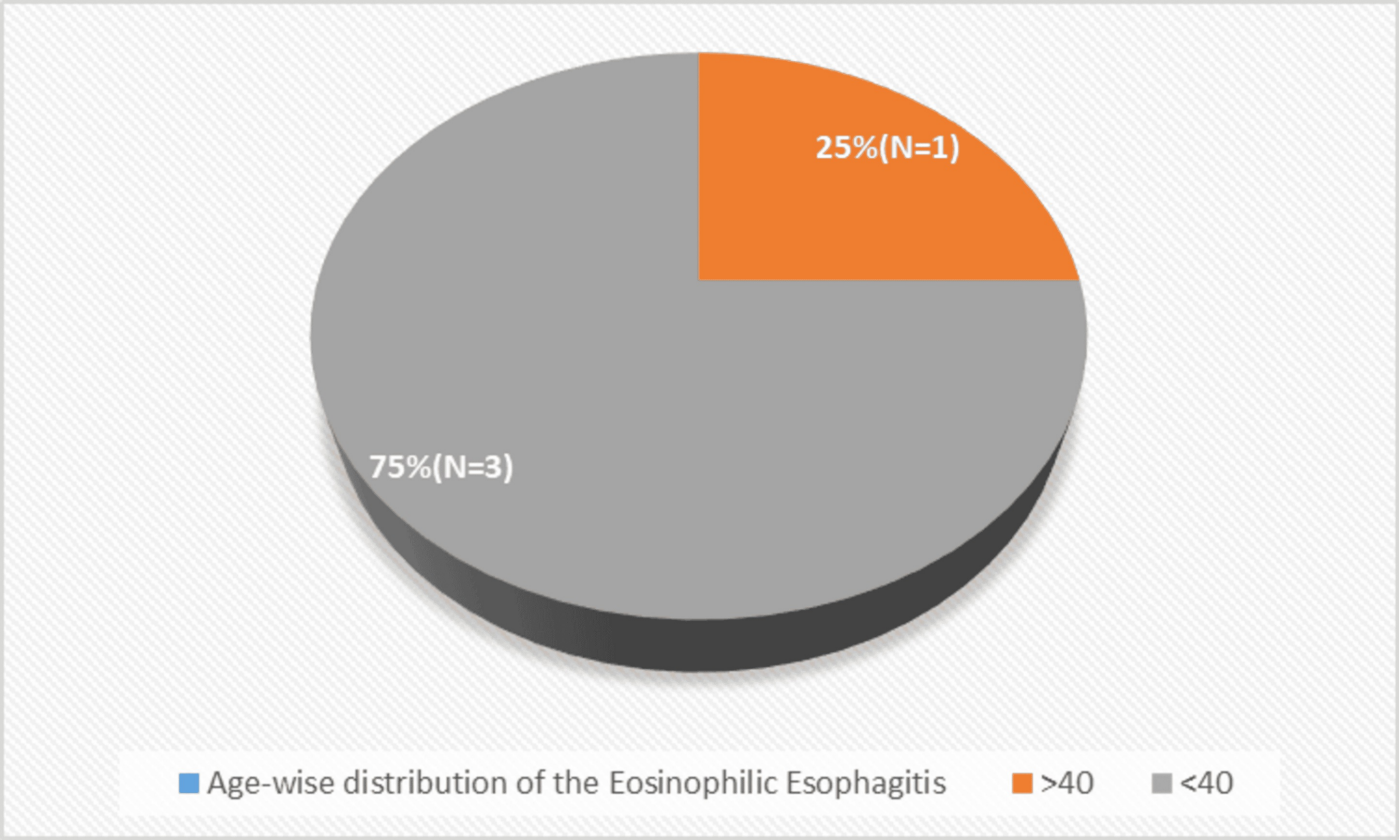
Age-wise distribution of the eosinophilic esophagitis

EoE was demonstrated predominantly (75%) in subjects above 40 years of age (Figure 5).

## Discussion

In our study, the prevalence of EoE in patients with symptoms of GER was 6.67%. To the best of our knowledge, this is the first study from Nepal to evaluate the hospital-based prevalence of EoE among patients with GER symptoms. Prasad et al. reported a 10-15% prevalence of EoE in patients suffering from dysphagia [14]. Similarly, a study by Veerappan et al. recognized EoE in 6.5% of cases among 400 patients undergoing EGD [16]. Various studies established a comparable prevalence of EoE, ranging between 2.4 and 6.6% [17-20]. The prevalence of EoE in patients with GER symptoms, refractory to PPIs, was found to be around 1-9% in different studies [21,22]. In a study done in North India of 190 consecutive patients with symptoms of GERD screened and esophageal biopsies available in 185 cases, six had EoE, suggesting a prevalence of 3.2% among patients with GERD [23]. In our study, the prevalence of EoE, purely in patients having GER symptoms, is 6.67% which is in concordance with the report by Veerappan et al., who reported EoE as. 6.5% in their study [16]. However, compared to a study done in North India by Baurah et al, our prevalence is almost double [23].

The genetic aspect of eosinophilic esophagitis (EoE) is indicated by its higher prevalence in male subjects. Research involving family histories, twin studies, and genome-wide association studies has consistently shown that the male-to-female ratio for EoE is approximately 3:1 [24]. However, in our study, the sex distribution of eosinophilic esophagitis was equal for both genders.

There is substantial evidence suggesting that EoE is primarily driven by the activity of type 2 helper T (Th2) cells and is largely triggered by food antigens. Several case series have consistently demonstrated that patients with EoE exhibit hypersensitivity to specific foods and environmental factors. These patients often respond positively to dietary exclusions of specific food antigens, and symptoms can relapse with the reintroduction of those same food antigens [25,26]. A personal or family history of atopic disorders: asthma, eczema, rhinitis, and anaphylactic food allergy, is common, and such conditions need therapy [27,28]. However, in our study, we did not find any personal or family history of atopic disorders, and we also could not demonstrate any food or environmental hypersensitivity or allergy.

A number of macroscopic endoscopic abnormalities have been associated with EoE. These classic endoscopic findings include multiple esophageal rings, longitudinal furrows, whitish plaques, and strictures. In the prospective study by Veerappan et al., the majority of EoE patients (72%) had one of these findings [16]. In our study, none of our patients with EoE had macroscopic endoscopic esophageal abnormalities consistent with EoE.

Limitations of the study

The present study has several limitations. First, there is a sampling bias, as EoE affects patients of all ages; however, we only included participants aged 18 and older. Second, none of the participants had a personal or family history of atopic disorders or hypersensitivity to food or environmental factors, which are commonly associated with EoE. Third, we did not include patients with dysphagia and food impaction. This has reduced the prevalence of EoE in our study, along with predominant normal endoscopic findings in 85% of the subjects. Fourth, we did not detect any increase in serum eosinophil counts or stool abnormalities in patients with EoE. To validate our findings, a prospective large-scale study examining the prevalence and predictive factors of EoE would be important.

## Conclusions

This study highlights a notable prevalence of eosinophilic esophagitis (EoE) among patients presenting with symptoms of gastroesophageal reflux (GER), even in the absence of visible endoscopic abnormalities. These findings underscore the value of routine esophageal biopsies, including in cases with macroscopically normal mucosa, to facilitate early diagnosis and appropriate management of EoE. The findings suggest the consideration of EoE as a diagnostic possibility in patients with GER symptoms in Nepal.
